# Reducing anxiety and enhancing confidence in paediatric patients through visual schedules

**DOI:** 10.4102/hsag.v30i0.2841

**Published:** 2025-02-05

**Authors:** Gert C. Koekemoer, Ariné Kuyler, Ensa Johnson, Karen van Zijl, Alta J. Terblanche, Khetsiwe P. Masuku, Juan Bornman

**Affiliations:** 1 Department of Health and Rehabilitation, Faculty of Medicine and Health Sciences, Stellenbosch University, Cape Town, South Africa; 2 Department of Inclusive Education, College of Education, University of South Africa, Pretoria, South Africa; 3 Department of Paediatrics, Zuid Afrikaans Hospital, Pretoria, South Africa; 4 Department of Speech Language Pathology and Audiology, Faculty of Humanities, University of the Witwatersrand, Johannesburg, South Africa

**Keywords:** visual schedules, paediatric person-centred care, communication support, augmentative and alternative communication, AAC, paediatric wellbeing, anxiety

## Abstract

Hospitalisation can induce anxiety and trauma in children, complicating their medical experience. Exploring the role of visual schedules as a supportive strategy reveals that these schedules can significantly reduce stress during medical procedures. By providing clear and structured guidance, visual schedules improve communication, foster patient engagement and create a sense of predictability, thereby enhancing the overall patient experience. When tailored to individual needs, they help children navigate healthcare settings with greater confidence. Effective staff training is crucial for successful implementation, ensuring that paediatric patients receive the maximum benefits.

Hospitalisation and medical interventions can create significant stress, fear and even trauma in children (Chicas et al. 2023). This distress may result in increased pain, developmental regression, separation anxiety (Ari et al. 2019), eating disturbances, enuresis (Batuman et al. 2016), sleep disorders (Romito et al. 2021) and reduced cooperation during medical procedures. Recent research indicates that incorporating images in medical procedures can be a valuable tool for explanation (Hafner et al. 2022). Images help to enhance understanding, promote dialogue between healthcare practitioners, paediatric patients and their families, and strengthen the therapeutic relationship.

Visual schedules are a type of visual support system that uses images to depict a sequence of events. As shown in Figure 1 (examples 1 to 6), these images can include real objects (e.g. syringes), photographs, graphic symbols, or written words. Visual schedules have strong evidence-based support in the field of education, particularly for children with disabilities (Liang et al. 2024; Thomas & Karuppali 2022). However, their use in hospital settings is relatively new. This commentary aims to explore the potential benefits and challenges of implementing visual schedules in this under-researched context.

**FIGURE 1:**
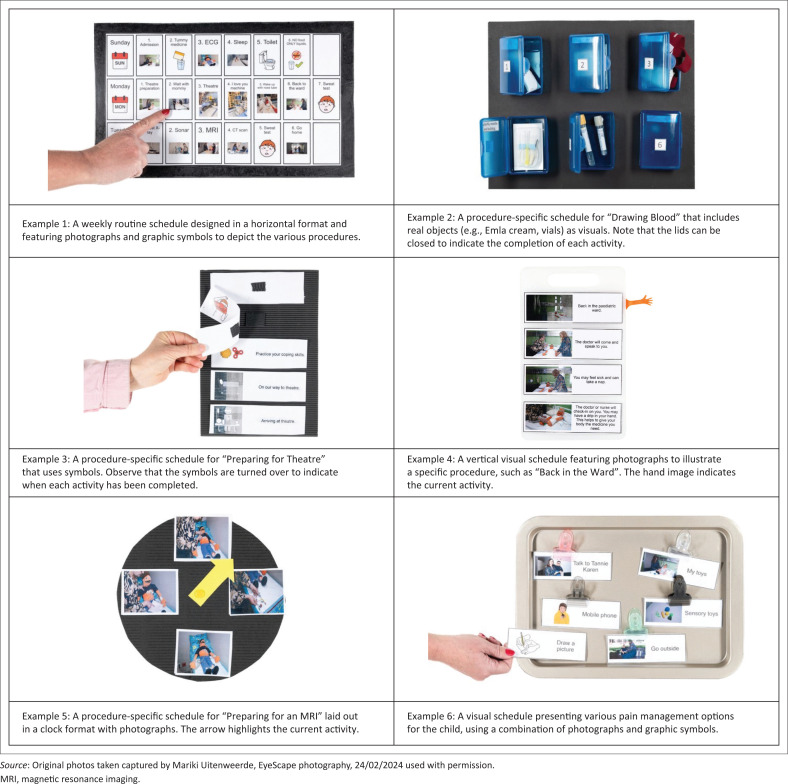
Examples of visual schedules.

## Benefits of visual schedules

In addition to traditional strategies such as verbal explanations or distraction techniques, several supportive approaches with a strong focus on person-centred care have been described in the recent literature for paediatric populations. These strategies include communicating treatment goals in an age-appropriate manner, educating and supporting the therapeutic triad (paediatric patient, family and medical team), considering the child’s emotions and striving to retain normal family life (Kardas 2024). One such strategy is the use of visual schedules, where images are employed to explain or demonstrate the steps involved in medical routines (e.g. blood draws, preparation for surgery). Because these schedules help children to understand and anticipate upcoming procedures, their potential anxiety and confusion are reduced. Since visual schedules show the sequence of events (such as visits to the doctor or specific medical tasks), they provide children with a sense of orientation, control and mastery in an otherwise overwhelming situation (Romito et al. 2021). Clarity about what to expect helps foster a sense of safety, enhances engagement, improves self-regulation and contributes to more effective care (Chicas et al. 2023). Moreover, visual schedules can improve communication between healthcare providers and paediatric patients in the manner descibed is consistent with person-centred care principles (Thunberg et al. 2022). The use of non-pharmacological complementary therapies such as visual schedules is now considered a gold standard in healthcare (Chebuhar et al. 2013). These therapies are part of a multimodal approach aimed at reducing anxiety, nausea, pain and other symptoms, and they are especially recognised for the value they add to nursing practice (Knoerr 2018).

Visual schedules vary in their specific functions. For instance, routine schedules are designed to outline daily activities during extended hospital stays, such as mealtimes, therapy sessions and visiting hours. These schedules help children grasp the day’s structure and prepare for transitions (Lang et al. 2011) (Example 1). After hospitalisation, visual schedules can assist with discharge planning as they outline the steps and actions needed for a smooth transition from hospital to home.

Procedure-specific visual schedules break down medical procedures into manageable steps. As illustrated in Example 2, a visual schedule for a blood draw might outline steps such as ‘sit in the chair’, ‘clean arm’, ‘insert needle’, and ‘apply bandage’ (Chebuhar et al. 2013). These schedules can also improve cooperation across various other hospital activities, including going to the operating theatre and receiving anaesthesia (Example 3), returning from surgery (Example 4), setting up an intravenous (IV) line and undergoing imaging procedures like x-rays or magnetic resonance imaging (MRIs) (Example 5). They are particularly useful in dynamic situations such as emergencies where they can be quickly adjusted to help children understand the situation and anticipate what will happen next (Royer et al. 2017). Visual schedules can also incorporate coping and pain management strategies such as deep breathing, distraction techniques and sensory toys, and in this way they can help reduce anxiety and support self-regulation during medical procedures (Chicas et al. 2023) (see Example 6).

## Design considerations

As explained in the previous section, visual schedules provide a clear and supportive way for children to understand and follow medical interventions. However, in line with the principles of person-centred care and to accommodate individual needs, they vary widely – depending on the child’s age, language and cognitive development (Light, McNaughton & Caron 2019; Macdonald, Corcoran & Herbert 2018). Designing these schedules therefore requires tailoring them to each child’s specific age, language, developmental and other needs. Appropriate designs range from simple charts with basic activities to detailed sequences of complex procedures. As indicated earlier, visual schedules use different images to convey information, and the images can represent practitioners, medical equipment or procedure rooms (see Figure 1). The images can be displayed on different surfaces such as poster boards or booklets, and they can be arranged in various layouts (e.g. horizontal, vertical, or circular). Images may be attached with Velcro for easy removal or repositioning, such as when activities change or when they have been completed. Depending on the child’s preferences, finished steps can be indicated by removing the particular image or turning the image over. Some children prefer a visual schedule that uses an arrow to point to the image that represents the current activity. When the activity has been completed, the child can move the arrow to the next image. This concrete action of moving the arrow to indicate progress (i.e. completion of an activity) enhances the child’s engagement and helps them experience a sense of accomplishment (Sippl 2023). Furthermore, providing enjoyable activities between less pleasant steps can boost motivation to complete the entire (sometimes stressful) medical routine, such as drawing blood (Bornman 2021).

## Implementation in paediatric care

Three key objectives guide the design of visual schedules: simplicity and clarity, personalisation and flexibility. Firstly, simplicity and clarity – which require using clear, easily recognisable symbols and minimal text (Romito et al. 2021) – are crucial. Whether employing line drawings or photographs, the images must be straightforward and easily identifiable. Secondly, personalisation – which should reflect the child’s age, developmental level, preferences and interests – is essential for making the schedule engaging. For example, if a child likes books or toy cars, these interests should be incorporated into their hospital schedule. Hodgetts, Zwaigenbaum and Nicholas (2019) reported greater engagement when visual schedules were personalised and co-constructed by the healthcare practitioner and child. Effective coping strategies for the child, such as pop-up books and sensory toys should also be included to help manage anxiety (Romito et al. 2021). Finally, flexibility is important to accommodate changes in the child’s condition or hospital routines. An easy-to-use ‘stick-on’ system with Velcro allows for quick adjustments to the schedule, thereby facilitating updates to the timetable or the inclusion of new activities (Hodgetts et al. 2019).

In the hospital setting, visual schedules enhance the consistency of care in several ways. By standardising the information provided to children and their families, they ensure that all staff members convey uniform messages about routines and expectations. These visual supports act as stable cues that reinforce verbal instructions and provide a reference point that improves comprehension and retention (Laxman et al. 2019). In addition, visual schedules facilitate smoother transitions between different hospital staff members by offering a reliable source of information about the patient’s activities. This consistency streamlines transitions between tasks handled by porters, ward nurses and operating room staff, making care more efficient and responsive (Batuman et al. 2016). Moreover, when combined with other mobile health tools such as scales and questionnaires for anxiety, nausea and pain, visual schedules help both paediatric patients and their parents and/or caregivers to communicate their experiences and monitor care satisfaction effectively.

## Potential challenges when implementing visual schedules

In order to ensure the effective implementation of visual schedules, stakeholders such as nurses, doctors and therapists must be trained. Their training might need to be spread over more than one session to ensure effectiveness, especially in cases where the activity depicted by the schedule is complex (Thomas & Karuppali 2022). Arranging this might be challenging in the hospital context, where staff have to cope with significant time constraints. Moreover, if the schedule requires a variety of materials (e.g. a combination of real objects and graphic symbols), it might have time and financial implications. (Nonetheless, visual schedules are still regarded as a low-cost solution and a feasible option in the South African context with its great variety of challenging socio-economic factors.)

An additional challenge was reported in a study conducted in a cosmopolitan hospital in Geneva, Switzerland. The researchers reported a potential risk of misinterpretation if healthcare practitioners and patients rely solely on visuals when interacting in multicultural context, as images are not universal (Hafner et al. 2022).

## Conclusion

To conclude, visual schedules have proven effective in improving paediatric patients’ experiences during hospital admission and medical procedures (Chicas et al. 2023; Romito et al. 2021). These visual aids do not only reduce children’s anxiety and foster their emotional safety, engagement and autonomy they also help the young patients to feel understood and in control. By offering clear, structured guidance, visual schedules empower children to navigate hospital routines with greater comfort and confidence (Hodgetts et al. 2019). Integrating these schedules into paediatric care creates a supportive environment that prioritises emotional well-being and enhances the child’s overall care experience.

Furthermore, visual schedules allow healthcare providers to focus on person-centred interventions, to standardise care practices, and to improve communication among staff members – all of which lead to smoother transitions and better patient outcomes (Hafner et al. 2022; Light et al. 2019). To maximise their effectiveness, visual schedules must be implemented consistently and proper staff training is therefore required to ensure their proper use.

Looking ahead, further research is needed to explore the long-term impact of visual schedules on patient outcomes as well as their adaptation for diverse paediatric populations, including for children with developmental delays or language barriers. Such studies could provide a deeper insight into how visual schedules can be tailored to meet the unique needs of different patient groups, and in this way person-centred care can be greatly improved.
